# Toward Patient-Centered Care: A Systematic Review of How to Ask Questions That Matter to Patients

**DOI:** 10.1097/MD.0000000000000120

**Published:** 2014-11-07

**Authors:** Alicia Rosenzveig, Ayse Kuspinar, Stella S. Daskalopoulou, Nancy E. Mayo

**Affiliations:** Division of Clinical Epidemiology (AR, NEM), McGill University Health Centre; School of Physical and Occupational Therapy (AK, NEM); and Department of Medicine (SSD), Faculty of Medicine, McGill University, Montreal, QC, Canada.

## Abstract

Clinicians rarely systematically document how their patients are feeling. Single item questions have been created to help obtain and monitor patient relevant outcomes, a requirement of patient-centered care.

The objective of this review was to identify the psychometric properties for single items related to health aspects that only the patient can report (health perception, stress, pain, fatigue, depression, anxiety, and sleep quality). A secondary objective was to create a bank of valid single items in a format suitable for use in clinical practice.

Data sources used were Ovid MEDLINE (1948 to May 2013), EMBASE (1960 to May 2013), and the Cumulative Index to Nursing and Allied Health Literature (1960 to May 2013).

For the study appraisal, 24 articles were systematically reviewed. A critical appraisal tool was used to determine the quality of articles.

Items were included if they were tested as single items, related to the construct, measured symptom severity, and referred to recent experiences.

The psychometric properties of each item were extracted. Validity and reliability was observed for the items when compared with clinical interviews or well-validated measures. The items for general health perception and anxiety showed weak to moderate strength correlations (*r* = 0.28–0.70). The depression and stress items showed good area under the receiver operating characteristic curve of 0.85 and 0.73–0.88, respectively, with high sensitivity and specificity. The fatigue item demonstrated a strong effect size and correlations up to *r* = 0.80. The 2 pain items and the sleep item showed high reliability (intraclass correlation coefficient [ICC] = 0.85, κ = 0.76, ICC = 0.9, respectively).

The search targeted articles about psychometric properties of single items. Articles that did not have this as the primary objective may have been missed. Furthermore, not all the articles included had the complete set of psychometric properties for each item.

There is sufficient evidence to warrant the use of single items in clinical practice. They are simple, easily implemented, and efficient and thus provide an alternative to multi-item questionnaires. To facilitate their use, the top performing items were combined into the visual analog health states, which provides a quick profile of how the patient is feeling. This information would be useful for regular long-term monitoring.

## INTRODUCTION

Forming a collaborative relationship between the patient and the clinician is the cornerstone of patient-centered care. This dialogue must focus on patients’ concerns, which cannot always be inferred from the clinical diagnosis and may need to be elicited through direct questioning. The literature suggests that patient-centered concerns, apart from survival, are symptoms, function, and health-related quality of life (HRQL).^1^

Although a profile of clinical health status requires comprehensive and systematic assessments of organ and system function, a profile of patient-centered outcomes would involve the administration of multiple items or questionnaires, the results from which need to be interpreted, monitored, and reinterpreted over time. Clinicians take a systematic approach to assessing clinical health status but are not commonly systematic in obtaining information on symptoms, functions, and HRQL—outcomes that matter to patients. There are a number of measures of HRQL, which have been used to improve quality of care in clinical practice^2^ with disappointing results.^3–10^ HRQL measures can provide tremendous insight into the matters of concern to patients and can track within-patient changes over time.^11^ Despite these benefits, HRQL measures are not yet widely used in patient care.^12^ One reason may be because clinicians face an uncertainty as to what the HRQL scores mean and how to apply the information. There is a lack of clarity about specific use of the information, including the ability to screen for problems, monitor progress over time, and facilitate models of patient-centered care.^13^ For this reason, a number of single items or questions have been developed to streamline the obtaining of this important information from patients.^14–17^ Collections are advantageous over multi-item questionnaires because they are simple and easy to implement,^18^ quick,^18^ less cognitively demanding,^19^ and can be stored as a reference to compare the score over time.^18,20^ To be useful in clinical practice, the single items must first relate strongly to the construct they have been developed to represent, as well as have the psychometric properties important for any measure. Thus, convergent or criterion validity (sensitivity and specificity if criterion is binary) is primordial for these single items, and evidence of reliability and responsiveness are additionally necessary.

Single-item patient-centered health outcome measures include specific performance tests (eg, walking speed) and asking questions to the patient directly. When questions relate to information on outcomes that only the patient can provide, these are termed patient-reported outcomes (PROs). PROs have been defined as “any report of the status of a patient’s health condition that comes directly from the patient, without interpretation of the patient’s response by a clinician or anyone else,”^21^ whereas non-PROs can be clinician-reported outcomes (ClinROs), observer-reported outcomes (ObsRO), or tests of performance (PerfROs). ClinROs arise from the results of a physical examination; the Apgar and the Preschool Respiratory Assessment Measure^22^ are examples. ObsROs are measures based on an observer (such as a family caregiver) assessing the patient’s behavior; examples include the Dementia Rating Scale,^23^ the brain impairment behavior scale,^24^ and the emotional behavior index form.^25^ The six-minute walk test,^26^ and the Barthel Index^27^ are examples of PerfROs, although the Barthel Index can also be completed by self-report or as an ObsRO. Although both ClinROs, ObsROs, and PerfROs can provide a physician with the status of a patient, there is no better way to improve patient-centered care than to increase the usage of PROs and ask patients directly about outcomes that matter to them.

The specific objective of this review was to identify the psychometric properties for single-PRO items through a search of the literature. A secondary objective was to create a bank of valid single items in a suitable format for clinical practice.

## METHODS

### Domains Under Study in Systematic Review

A systematic review was carried out. Specific domains were chosen based on PROs meaningful for improved patient-centered care and that were not assessable through physical examination or performance testing. Seven domains were selected as meeting these criteria, also because they are often queried directly or indirectly within a health care encounter: general health perception, stress, pain, fatigue, depression, anxiety, and sleep.

### Search Methods for Identification of Studies

Articles were identified by searching the following databases: Ovid MEDLINE (1948 to May 2013), EMBASE (1960 to May 2013), and the Cumulative Index to Nursing and Allied Health Literature (1960 to May 2013). The same principle was used to search each database for each domain, which included the following terms: depression/mood, fatigue/energy, anxiety, sleep/sleep quality, pain, stress/distress, and self-rated health/general health perception. Each of these terms were combined with the following terms: validity OR concurrent validity OR discriminant validity OR construct validity OR criterion-related validity OR validation studies OR instrument validation OR known-groups OR discriminant analysis OR reliability OR intrarater reliability OR test-retest reliability OR interrater reliability OR intraclass correlation coefficient OR kappa statistic OR minimally significant OR minimal detectable OR psychometrics OR responsiveness OR minimally important change OR meaningful change OR minimal detectable OR minimally significant. Finally, all of these search terms were combined with the term single-item using the Boolean operator AND.

This literature review was supplemented by known articles of valid single items: the visual analog mood scales (VAMS)^28^ and the global items in the patient-reported outcomes measurement information system (PROMIS).^29^

As this was a literature review and did not involve recruitment of patients, ethical approval was not necessary.

### Article Screening and Data Extraction

Only full publications in peer-reviewed journals were considered. Unpublished data, abstracts, grey literature, and studies published in languages other than English or French were excluded. All study designs (randomized controlled trials, cross-sectional studies, etc.) and health conditions were included. The study population was restricted to adults.

Two authors (A.R. and A.K.) independently screened the citations and abstracts identified in the search and amalgamated them into Reference Manager 12, Thomson Reuters. Based on the abstracts, duplicate or irrelevant articles were excluded, as were full text articles that did not meet inclusion criteria.

### Item Selection and Inclusion Criteria

Items were included in the systematic review based on the following criteria:

1. developed as a single- or stand-alone item or had been tested for this intent even if it had originally been part of a multi-item measure;
2. appeared to relate strongly to the construct it had been developed to represent;
3. measured severity, not impact; and
4. referred to recent experience (current or past week).

The multidimensionality of measuring symptoms can create challenges as they can vary in severity, duration, frequency, and impact.^30^ We therefore excluded items referring to the impact because it can change as people modify their activities and lifestyles,^30^ reporting low impact because of curtailment of activities without there being actual change in symptom intensity, duration, or frequency. Thus, it was also important to choose items where the wording directly tapped the construct rather than its consequences. For example, an item referring to depression needs to use words referring to elements of mood and not to the degree of engagement in activities or roles. Additionally, we favored items assessing the current time frame and not those requiring historical averaging (eg, past 2 weeks, past month). Ultimately, items were selected if deemed useful in a clinical setting, were quick to administer, and yielded answers meaningful for both clinicians and patients.

### Data Extraction and Quality Assessment

A data extraction form was created to identify the study population (age, sex, and targeted health condition), study characteristics (country where the study took place, recruitment method, language, sample size), external reference, and psychometric parameters (validity, reliability, and responsiveness). Any disagreements on the eligibility of a study were resolved by consensus.

The quality of the articles chosen for inclusion was determined by using a 13-item critical appraisal tool developed specifically to assess psychometric properties for items used in clinical practice.^31^ The items are listed in Appendix 1. Of the 13 items, 4 were for articles assessing reliability, 4 were for validity studies, and the remaining 5 items were for both.^31^ Two authors (A.R. and A.K.) independently screened each article with the critical appraisal tool. With each of the 13 items, the critical appraisal tool gives a justification as to why the criterion should be evaluated in articles and a scoring rubric to help decide whether the article met the criteria or not. The quality assessments for each article were compared and discrepancies resolved. Authors answered either yes/no/not applicable for each of the 13 items and scores ≥80% for the article was considered good quality (Table 1).

**TABLE 1:**
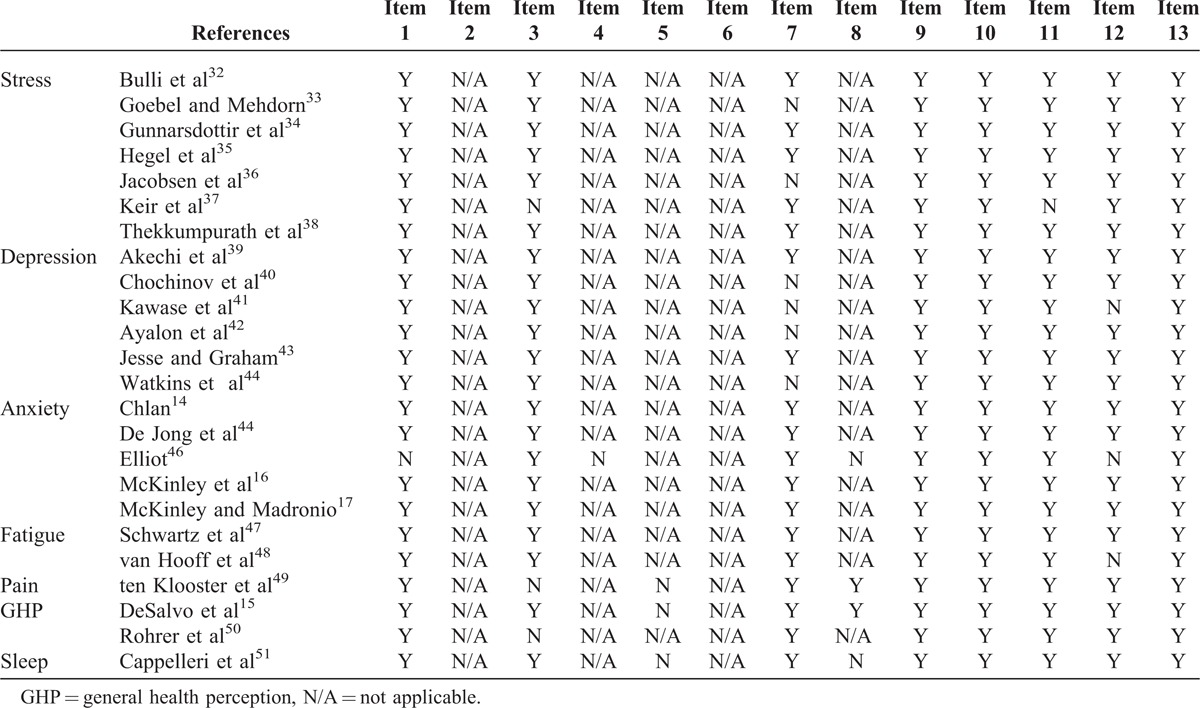
Quality Assessment of Articles

Data on the psychometric properties of validity, including accuracy parameters, reliability, and responsiveness to change were extracted from the articles included in the review.

### Psychometric Properties Evaluated

Validity refers to the extent to which an item is measuring the construct it claims to measure.^52^ The item can be compared to a diagnosis, or “gold standard,”^53^ or an already validated and reliable measure of the construct.^53^ Validity is assessed by parameters such as the area under the receiver operating characteristics (ROC) curve, and correlation coefficients, such as Pearson *r* and Spearman ρ, are the parameters used to quantify validity.^54^ The area under the ROC is calculated from the plot of the sensitivity and 1-specificity at each cut-point of the measure.^53^ For these, the parameters closest to 1.0 have the highest validity.

Test-retest reliability assesses the stability of patients’ responses over time, given that the patients have not changed.^52,54,55^ Three parameters were commonly reported: the intraclass correlation coefficient (ICC) for continuous variables; correlation coefficients, mainly Pearson *r*; and Cohen κ for dichotomous or ordinal variables, as well as weighted κ for ordinal variables.^53^

Responsiveness, which can be considered an aspect of validity,^53^ detects change over time.^52^ If patients have changed on the construct being measured, their scores should reflect this change accordingly. Responsiveness of an item is determined by how well it captures this change in status.^53^ Responsiveness is commonly reported using the effect size (ES). For all of the parameters mentioned, the closer the value was to 1.0, the more acceptable the psychometric property was for the single item.

### Psychometric Property Cut-Offs for Interpretation

To interpret the measurement properties extracted from the articles, standardized criteria was used for the values of validity, reliability, and area under the ROC curve.

Several authors have strength criteria for validity. However, it is all dependent on context and here we chose to use Cohen κ, where a correlation of 0.20 was small, 0.50 was moderate, and 0.80 was strong.^56^ For interpreting the area under the ROC curve, which ranges from 0 to 1.0 (perfect prediction), 0.5 was poor (equivalent to predicting by flipping a coin), >0.7 indicated acceptable predictions, and >0.8 excellent predictions.^57^ For κ, (weighted or not) values of 0.75 or greater were considered excellent agreement.^58^ As for responsiveness, an ES of 0.2 was considered weak, 0.5 moderate, and 0.8 strong.^56^

## RESULTS

**FIGURE 1:**
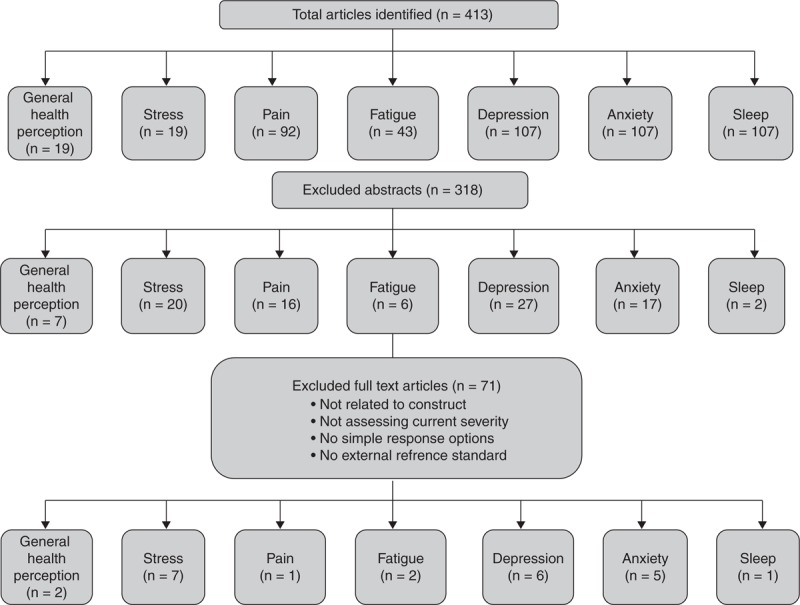
Flowchart of the literature search.

Figure 1 presents the flowchart of the literature search. The 7 constructs were searched and a total of 773 articles were found. After duplicates were removed (n = 360), a total of 413 abstracts were identified through the different databases. Of these, 318 abstracts were excluded because they were irrelevant, did not include a single-item PRO, or were unpublished data. Remaining articles were then evaluated for eligibility. Seventy-one articles were excluded upon full text review because they did not meet the single-item inclusion criteria (ie, not relating to the construct, not assessing symptom severity, no simple response options), whereas other articles were excluded because of no external reference standard. A total of 24 eligible articles were reviewed systematically. The literature on the PROMIS global items was not included as they had been validated against another single-item PRO question, namely, the classifiers of the EQ-5D.^29^ Also, the VAMS items were not included because they were validated as a total score and not as standalone items.^28^

The 24 studies^14–17,50–51^ included in our review were from countries all over the globe, including but not limited to Japan, the USA, Israel, several European countries, and Canada. The main study populations were patients with cancer,^32–37,39–41,47^ stroke,^44^ and myocardial infarction,^45,46^ and seniors in primary care settings.^42^ The sample sizes ranged from as low^50^ as 34 to as high as 1493.^51^

### General Health Perception

One item for general health perception was found from 2 articles. “How is your health in general?”^15,50^ showed weak to moderate strength correlations (*r* = 0.37–0.66) when compared with the Short Form-12, Thomson Reuters, and a test–retest reliability of ICC = 0.69 (Table 2). The articles^59^ had good methodological quality.

**TABLE 2:**
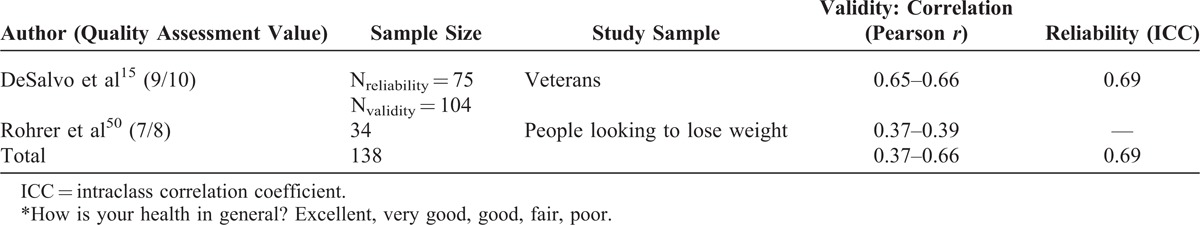
General Health Perception^∗^

### Stress

Stress was evaluated in 7 articles using the distress thermometer (DT), a visual analog scale (VAS) (Table 3).^32–37^ This item was compared with several self-reported questionnaires, for example, the Patient Health Questionnaire. With DT cut-off scores ranging from 3 to 8, the area under the ROC ranged from fair to good (AUC = 0.73–0.88).^32–37^ The item also showed a range of correlational strengths, although most were weak to moderate (*r* = 0.23–0.60).^32–37^ All but 1 of the articles were of good quality.^37^

**TABLE 3:**
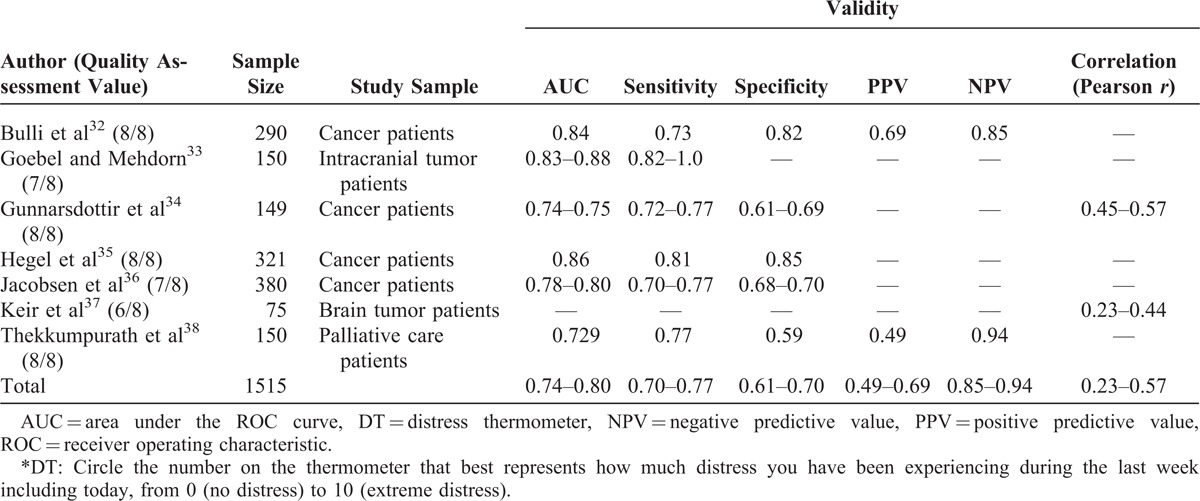
Stress^∗^

### Pain

Two pain single items from 1 article were included in the review (Table 4). The first was a graphic rating scale (GRS) of pain severity, and the second was a verbal rating scale (VRS) where the response option is categorical (none to severe).^49^ Both the GRS and VRS showed acceptable test–retest reliability (ICC = 0.85, κ = 0.76, respectively) and ranged from weak to moderate correlations (*r *= 0.24–0.65) when compared with other pain measures.^49^ The article had good methodological quality.

**TABLE 4:**
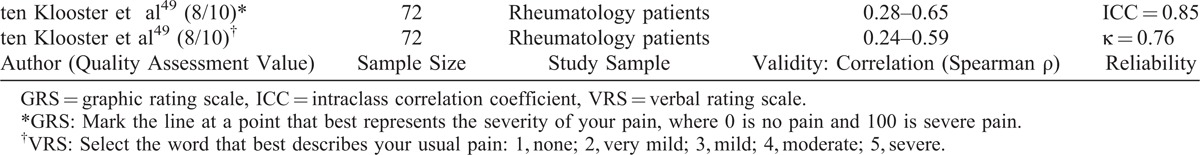
Pain

### Fatigue

Two items from 2 articles showed suitable clinical application (Table 5). Both of the items used the VAS metric, but different pegs: 0 (no fatigue) to 10 (greatest possible fatigue)^47^ and 1 (not at all fatigued) to 10 (extremely fatigued).^48^ “What is your current level of fatigue today?”^47^ had a large effect size (0.78) and “How fatigued do you currently feel?”^48^ showed weak to strong correlations (*r* = 0.16–0.80) when compared with self-reported measures, such as the daily fatigue item in the Profile of Mood States. One of the articles demonstrated good discriminant validity (*r* = −0.02 to 0.10)^48^ when compared against constructs unrelated to fatigue. Both the articles showed good methodological quality.

**TABLE 5:**
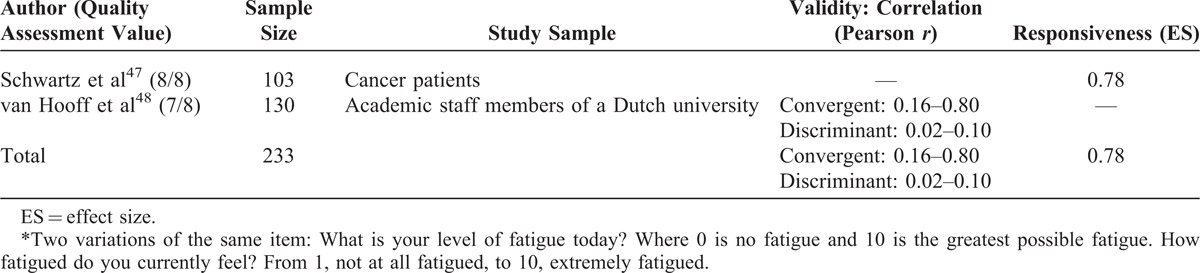
Fatigue^∗^

### Depression

Three variations of single items from 6 articles were included for depression single item: “Are you depressed?”^39–41^; “Do you think you suffer from depression?”^42^; and “Are you often sad or depressed?”^43,44^ (Table 6). The response options were binary (yes/no). The items, compared with structured clinical interviews and validated depression questionnaires, showed good accuracy with the area under the ROC curve of 0.85 and large sensitivities and specificities (sensitivity = 0.42–1.0, specificity = 0.60–1.0).^39–44^ Of the 6 depression articles, 5 had good methodological quality, whereas 1 of them had moderate methodological quality.^41^

**TABLE 6:**
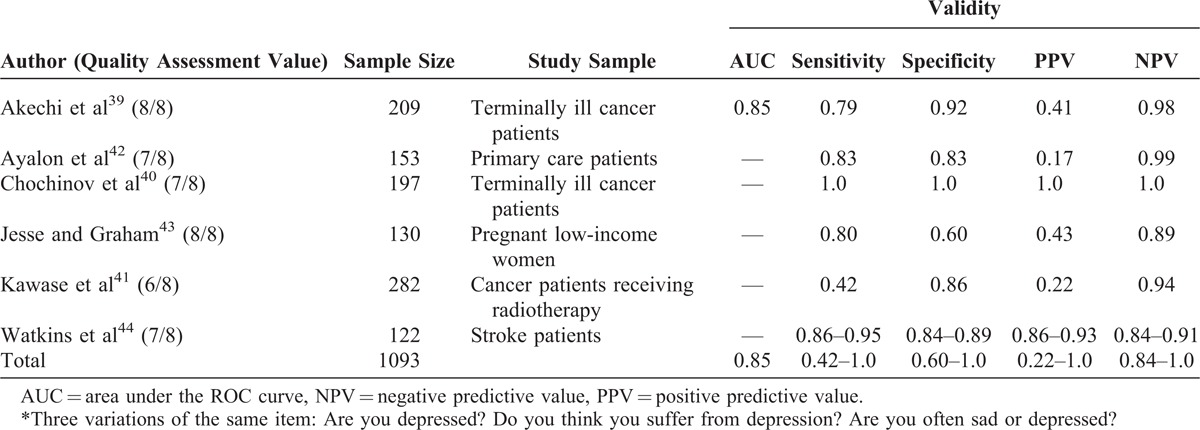
Depression^∗^

### Anxiety

Two items from 5 articles were included to assess anxiety: a VAS^14,45,46^ and a face anxiety scale (FAS) (Table 7).^16,17^ When compared with anxiety questionnaires, the VAS showed a range of correlations from weak to moderate strength (*r *= 0.28–0.70).^14,45,46^ The FAS showed moderate strength correlations when compared with interviews and self-reported anxiety scales (*r *= 0.64–0.70).^16,17^ Four of the 5 articles were of good quality,^14,16,17,45^ whereas 1 showed moderate methodological quality.^46^

**TABLE 7:**
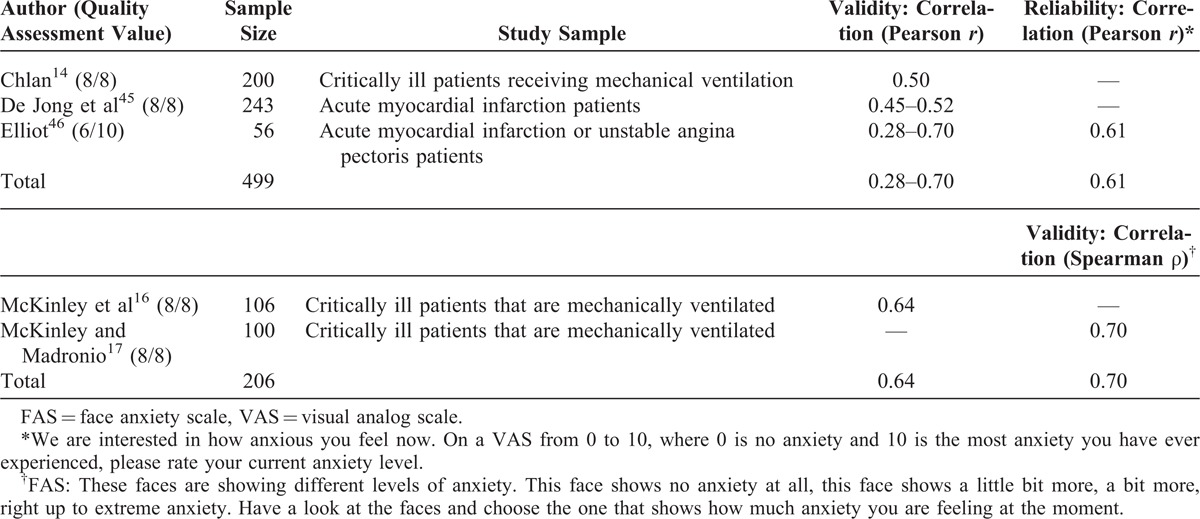
Anxiety

### Sleep

The Sleep quality scale was the single item included^51^ (Table 8). This item showed acceptable test–retest reliability (ICC = 0.9); however, Pearson correlations were weak when compared with different subscales of the Medical Outcomes Study sleep scale domains (*r* = 0.11–0.45). The article had good methodological quality.

**TABLE 8:**
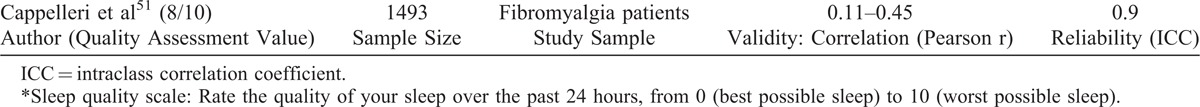
Sleep^∗^

### VAHS: How Are You Today?

**FIGURE 2:**
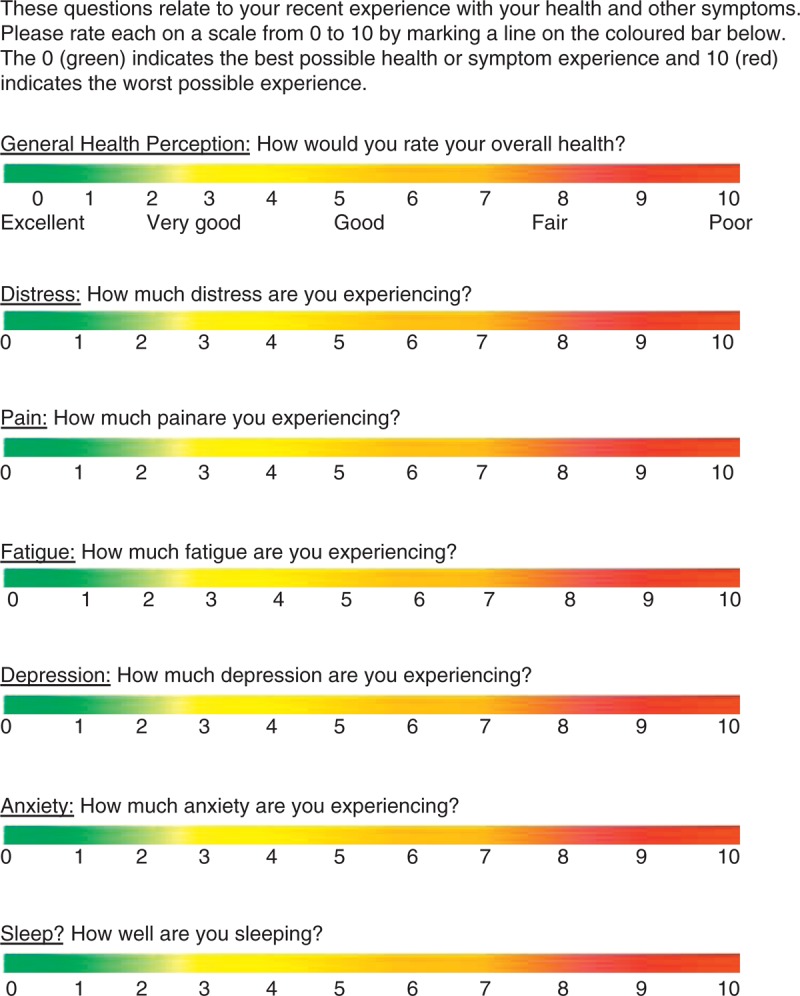
How are you today? Visual analog health states.

Although there was a range of values for psychometric properties, for each domain assessed, there was at least 1 study showing strong relationship with the intended construct, all studies supporting a positive relationship (Figure 2). There is sufficient validity for these single items to warrant asking them in clinical practice. However, no one response set was tested. The most frequent option was the VAS. The VAS is widely used as a metric and has had a presence in the literature for almost a century.^60,61^ By compiling the 7 single items identified in this literature review and using the VAS metric, we have created the visual analog health state (VAHS) form, which is presented in Figure 2.

## DISCUSSION

The purpose of this review was to provide a clinically relevant bank of reliable and valid single-item outcome measures that could be used to efficiently and effectively structure the patient/clinician dialogue around patient-centered outcomes. To achieve this, the psychometric properties of the single items were reviewed and there was support for their validity as appropriate questions to ask in a clinical setting.

There are several advantages associated with the use of single items over multi-item questionnaires. They are simple and easy to implement, more time efficient,^18^ inexpensive,^20^ and can be more appropriate in certain patient populations.^19^ Conversely, questionnaires that have multiple items can be time-consuming for patients as well as require significant concentration and attention. Regardless of the disease population, single items can act in the best interest of patients in that they respect patients’ time and can access valuable data without a cognitively demanding multi-item questionnaire.^19^ These items can be used to monitor changes in health states through the review of the single-item score over time.^18,20^ Additionally, the clinical utility of single items serves to maximize the time spent on interviews or physical evaluations and complement the overall assessment without interfering with the lengthy routine of clinical process.^18^ Furthermore, there are specific patient populations in which single-item measures are the only appropriate option, such as critically ill patients, where it can be difficult to evaluate PROs with interviews or lengthy questionnaires.^19^

The literature supports the usefulness of single items. Our search revealed several examples of valid single-item variations for use in clinical settings. Specifically, the single item for general health perception was significantly correlated with tumor necrosis factor-α, interleukin-6, and C-reactive protein levels for all age groups, where worse health correlated with higher levels of the inflammatory cytokines^62–64^ as well as other circulating biomarkers.^65^ A single question added into a clinical encounter can provide both a self-reported psychological and biological marker of a patient’s health status.^62^ If the patient reports poor self-rated health, the physician can use this as an indicator of a potential underlying illness that may otherwise go unnoticed.^62^

Asking relevant questions is part of etiquette-based medicine.^66^ Eliciting patients’ concerns can help emphasize feelings of respect and present the physician as more courteous and humane.^66,67^ Some of these behaviors are as simple as introducing yourself and shaking the patient’s hand when entering the room.^66^ This greatly increases patient satisfaction, reduces anxiety, and increases adherence to medication or treatment^68,69^; however, these behaviors are not always performed.^70^ An important component of etiquette-based medicine is the use of open questions rather than yes/no questions to elicit feelings.^66^ Optimal behavior would also include paying attention, reporting the answers, and using them for future comparison. We propose that by asking patients about the 7 domains mentioned, patient-centered care could be improved. The VAHS gives a quick profile of a patient’s PRO health state, which provides useful information about the patient at the present time and can be used as a reference point for monitoring during the course of treatment.

A limitation in our review is that the identification of articles validating single items was not uncomplicated because there is no consistent way in which the information is indexed. As a result, articles were likely missed. Enhancing the article selection with known literature was an attempt to fill some gaps. Hence, we looked at the literature on VAMS^28^ and PROMIS,^29^ but it was not retained for the analysis because the method of cross-validation did not meet our criteria. These 2 sources, however, support the value in using single items in general.

Furthermore, we specifically targeted articles on the psychometric properties of these measures. There may have been additional sources that provided information on validity and reliability but not as the primary objective of the study. As well, there are limitations when using only 1 item to assess complicated outcomes. Multi-item questionnaires have more consistency, are less susceptible to bias,^18^ and may provide more information than just 1 self-reported item. There are advantages and disadvantages to both single and multi-item PRO questionnaires, and there is evidence for the usefulness of both. It is important to realize the contextual and situational uses for each one.

We chose the VAS as the response set for the VAHS. Psychometric properties of the VAS have been frequently studied for mood and pain.^69^ Validity shows correlation strengths ranging from moderate to strong for pain,^72–74^ and weak to moderate for mood.^75^ The VAS demonstrates an ability to discern small decreases in pain in a clinical setting,^74,76^ as well as discriminate between different levels of pain intensity.^76^ Although quick and easily administered, there are limitations to its use. The VAS metric can vary with experience,^71^ for example, a maximal value of pain for an individual can change if, between the 2 VAS time-point measurements, the patient has a painful experience.^71^ Despite these limitations, the VAS has demonstrated strong evidence of validity, simplicity, and clinical usefulness in a prospective manner. Alternatively, a Likert scale with verbal responses (not at all, a little bit, somewhat, quite a bit, very much) is a common method for scoring single items,^29^ but the interpretation of the qualifiers may not be the same across people, health conditions, and languages.

In conclusion, we recommend that clinicians use these validated items in their clinical practice to enhance patient-centered care and permit tracking of a patient’s progress over time. Future research should focus on evaluating the impact of using such a reporting system on patient and clinician satisfaction, as well as adherence to treatment.

## APPENDIX 1

### Quality Assessment of Articles

1. Item 1: If human subjects were used, did the authors give a detailed description of the sample of subjects used to perform the (index) test?
2. Item 2: Did the authors clarify the qualification, or competence of the rater(s) who performed the (index) test?
3. Item 3: Was the reference standard explained?
4. Item 4: If interrater reliability was tested, were raters blinded to the findings of other raters?
5. Item 5: If intrarater reliability was tested, were raters blinded to their own prior findings of the test under evaluation?
6. Item 6: Was the order of examination varied?
7. Item 7: If human participants were used, was the time period between the reference standard and the index test short enough to be reasonably sure that the target condition did not change between the 2 tests?
8. Item 8: Was the stability (or theoretical stability) of the variable being measured taken into account when determining the suitability of the time interval between repeated measures?
9. Item 9: Was the reference standard independent to the index test?
10. Item 10: Was the execution of the (index) test described in sufficient detail to permit replication of the test?
11. Item 11: Was the execution of the reference standard described in sufficient detail to permit its replication?
12. Item 12: Were withdrawals from the study explained?
13. Item 13: Were the statistical methods appropriate for the purpose of the study?
